# Molecular Determinants of Ca_v_1.2 Calcium Channel Inactivation

**DOI:** 10.5402/2012/691341

**Published:** 2012-10-17

**Authors:** Nikolai M. Soldatov

**Affiliations:** Humgenex Inc., Kensington, MD 20895, USA

## Abstract

Voltage-gated L-type Ca_v_1.2 calcium channels couple membrane depolarization to transient increase in cytoplasmic free Ca^2+^ concentration that initiates a number of essential cellular functions including cardiac and vascular muscle contraction, gene expression, neuronal plasticity, and exocytosis. Inactivation or spontaneous termination of the calcium current through Ca_v_1.2 is a critical step in regulation of these processes. The pathophysiological significance of this process is manifested in hypertension, heart failure, arrhythmia, and a number of other diseases where acceleration of the calcium current decay should present a benefit function. The central issue of this paper is the inactivation of the Ca_v_1.2 calcium channel mediated by multiple determinants.

## 1. Introduction

The voltage-gated inward Ca^2+^ current (*I*_Ca_) is a common mechanism of transient increase in the cytoplasmic free Ca^2+^ concentration triggered by cell depolarization. This form of Ca^2+^ signaling activates essential cellular processes including cardiac contraction [1], regulation of a smooth muscle tone [2], gene expression [3], synaptic plasticity [4] and exocytosis [5]. Complete and rapid termination of Ca^2+^ influx is mediated by an intricate mechanism of spontaneous calcium channel inactivation, which is crucial for preventing Ca^2+^ overloading of the cell during action potentials and restoration of the resting sub-*μ*M cytoplasmic free Ca^2+^ concentration [6]. This paper will focus on the molecular basis and multiple determinants of the Ca_v_1.2 calcium channel inactivation.

## 2. Ca_v_1.2: Challenges and Solutions

### 2.1. Molecular Complexity

**Figure 1:**
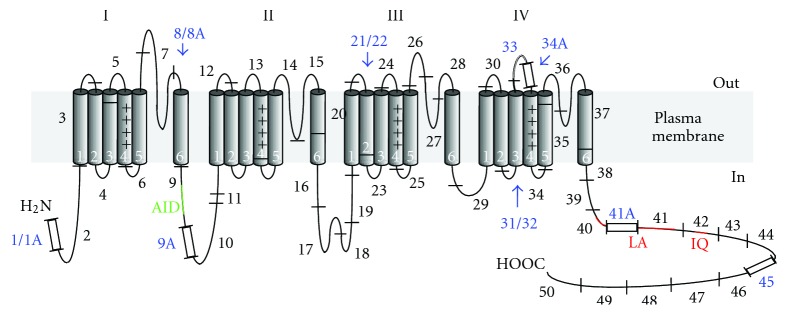
Transmembrane topology of the *α*_1C_ subunit. To illustrate the sites of molecular diversity, the polypeptide sequence is schematically segmented according to the CACNA1C genomic map [14] and the corresponding invariant (black) and alternative (blue) exons are outlined by black bars and numbered (1–50). Four regions of homology (I–IV), each composed of 6 transmembrane segments (numbered), are believed to be folded around the central pore. *α*-interaction domain (AID) of a constitutive *β*-binding site is shown in green. LA and IQ motifs (red) constitute calmodulin-binding domain (CBD).

The Ca_v_1.2 calcium channel is an oligomeric complex composed of the *α*_1C_, *α*_2_ *δ*, and *β* subunits [7, 8]. The ion channel pore is formed by the *α*_1C_ peptide (Figure 1) that is encoded by the *CACNA1C* gene. The auxiliary *β* and *α*_2_ *δ* subunits are essential for the functional expression and plasma membrane (PM) targeting of the channel [9, 10]. They exist in multiple genomic isoforms generated by four *CACNB* genes (*CACNB*1–4) and three *CACNA2D* genes (*CACNA2D1*–3). All three subunits are subject to alternative splicing. Adding to the complexity of the Ca_v_1.2 molecular organization, *β* subunits tend to oligomerize [11]. All together, genomic variability, alternative splicing, and hetero-oligomerization generate a plethora of Ca_v_1.2 splice variants that are expressed in cells in species-, tissue-, and developmental-dependent manner, while the change of their fine balance may have significant pathophysiological consequences [12, 13]. 

### 2.2. Challenges in the Selection of the Host Cell

Naturally occurring diversity of Ca_v_1.2 complicates the interpretation of data obtained from native cells, let alone the single channel data. This underlies the importance of Ca_v_1.2 research in recombinant expression systems where the molecular composition of the channel and the structure of its constituents are predefined. However, this experimental approach encountered the major problem of the selection of an appropriate host cell.

 Most of the studies of calcium channels were carried out using HEK293 cells. These cells provide high expression efficiency of recombinant Ca^2+^ channels but, unfortunately, contain endogenous calcium channels exhibiting Ca^2+^ currents up to 3 pA/pF [15, 16]. Thus, HEK293 cells allow for the adequate study of recombinant Ca^2+^ channels only when the amplitude of the current is large enough to ignore the contribution of the endogenous channels. Correct assessment of the functional determinants of Ca^2+^ channels, however, requires the use of host cells that are completely free of endogenous Ca^2+^ channel subunits. COS1 or COS7 cells suit this requirement well because they generate no appreciable calcium current, do not contain endogenous Ca^2+^ channel subunits or their precursors, and show no induction of endogenous Ca_v_1.2 subunits in response to the expression of the recombinant ones [17, 18]. Kinetics parameters and voltage dependence of activation and inactivation of the Ca_v_1.2 channel currents measured in COS1 cells are consistent with data obtained in other expression systems [19]. An important advantage of COS cells is their relatively slow division rate that allows for better control over efficiency of expression and assembly of the Ca_v_1.2 channel subunits of different size.

### 2.3. Problems of Fluorescent Labeling and Measurement

 Fusion of GFP-like fluorophores to the N- and/or C-termini of the recombinant *α*_1C_ or to the N-terminus of *β* does not markedly change the electrophysiological properties of the expressed channels, enables the application of fluorescent and FRET (fluorescent resonance energy transfer) microscopy to the study of subcellular distribution and assembly of Ca_v_1.2 as well as intricate aspects of molecular architecture and dynamics of the channel. The channel retains major electrophysiological characteristics unchanged when the *α*_1C_ C-terminal sequence encoded by distal exons 46–50 (Figure 1, residues 1833–2138 in *α*_1C,77_) is replaced by ECFP. However, *α*_1C_ fused by its N- or/and C-termini to EYFP is highly sensitive to photobleaching that irreversibly inactivates it. Known as fluorophore-assisted light inactivation (FALI), this interesting property limits the applicability of acceptor photobleaching for the measurements of FRET in Ca_v_1.2 because of uncertainty in the functional state of the channel [20]. However, the ratiometric analysis of corrected FRET between the fluorophores, fused to the tails of the *α*_1C_ and/or *β* subunits, reflects the reversible state-dependent structural rearrangements of the channel induced by the changes of transmembrane voltage under patch clamp [19, 21].

### 2.4. Recombinant Ca_v_1.2: What Does It Need for Functional Expression and How Does It Appear?

**Figure 2:**
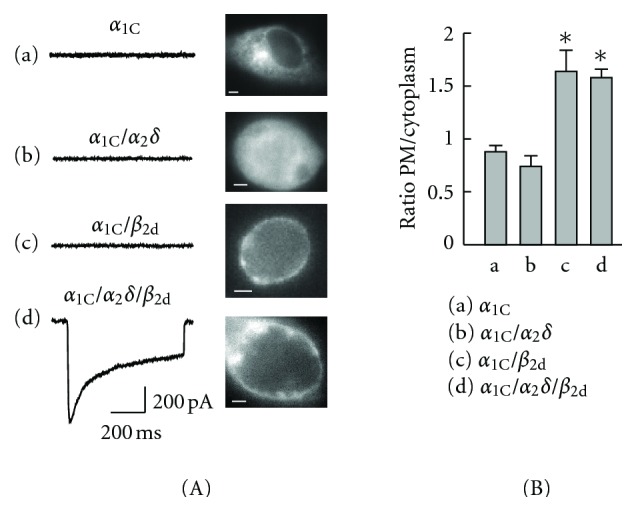
Role of the Ca_v_1.2 auxiliary subunits. (A) Epifluorescent images of the expressing COS1 cells showing distribution of EYFP_N_-*α*_1C_ obtained with the YFP filter (scaling bars, 4 *μ*m) and traces of the maximum calcium current recorded in response to 600 ms steps to +30 mV from the holding potential *V*_*h*_ = −90 mV (left). (B) Relative distribution of EYFP_N_-*α*_1C_ in the plasma membrane (PM) over the cytoplasm in the absence (a) or presence of *α*_2_ *δ* (b), *β*_2d_ (c), or *α*_2_ *δ* + *β*_2d_ (d). The ratio of fluorescence intensity in PM over the area underneath PM was averaged after background subtraction in each cell. The ratio less than 1.0 indicates lack of significant PM targeting by *α*_1C_. ∗*P* < 0.05 [18].

 Typical properties of a “wild-type” recombinant Ca_v_1.2 are illustrated in Figure 2(A) using an example of the ubiquitous human *α*_1C,77_ isoform (GenBank no. z34815). When the EYFP-labeled *α*_1C_ was expressed in COS1 cells alone, the fluorescent-tagged channel protein was diffusely distributed over the cytoplasm and did not generate measurable calcium current (Figure 2(A), panel a). The quantitative analysis of distribution of *α*_1C_ between PM and the cytoplasm (Figure 2(B)) [18] confirmed lack of significant PM targeting by *α*_1C_ independently on the presence of *α*_2_ *δ* (bars a and b). Expression of Ca_v_ *β* in the absence of *α*_2_ *δ* stimulated PM targeting of *α*_1C_, but the channel remained silent (Figure 2(A), panel c) unless *α*_2_ *δ* was coexpressed (panel d). Thus, *β* and *α*_2_ *δ* subunits are sufficient for the functional channel; under these experimental conditions, *β* subunits stimulate PM targeting of the channel complex and, in the presence of *α*_2_ *δ*, facilitate voltage gating of the Ca_v_1.2 channel.

 The shape and appearance of the peak calcium current shown in Figure 2(A) (panel d) is quite typical for the *β*_2_-modulated Ca_v_1.2 [22]. Its major features include the relatively slow rate of *I*_Ca_ decay and a large fraction of the sustained *I*_Ca_ remaining the end of the depolarizing pulse [18]. It is clear that during long-lasting action potentials such properties may lead to pathogenic calcium overload of the cell if it is not balanced by robust compensatory mechanisms. It was the ultimate role of Ca_v_1.2 in defining the duration of the action potential in cardiac cells that triggered the research and development of calcium channel blockers, a class of drugs that by now has a billion dollar market. It is this role of Ca_v_1.2 that stimulates the current interest to the identification of molecular determinants of Ca_v_1.2 inactivation in hopes of finding more specific and more effective drugs.

### 2.5. Last but Not Least a Complication: Ca_v_1.2 Clustering

A single ventricular myocyte contains ~300,000 Ca_v_1.2 channels, but only ~3% of the channels are open at peak *I*_Ca_ [23]. Contrary to the popular belief, Ca_v_1.2 channels are not evenly distributed over the plasma membrane. In native neuronal [24–26] and cardiac muscle cells [27–29] they form large clusters. Single-molecule imaging of the functional recombinant EYFP_N_-*α*_1C_/*β*_2a_/*α*_2_ *δ* channels expressed in HEK293 cells revealed clusters composed of ~40 channels that were mobile in the plasma membrane [30]. Both the fluorescence correlation spectroscopy and fluorescence recovery after photobleaching experiments yielded a lateral diffusion constant of *D*_lat_ ≈ 0.1 *μ*m^2^/s. The functional significance of the Ca_v_1.2 clusters mobility is not clear. It is believed that in cardiac muscle cells such mobility may be restrained by interactions with other proteins, for example, ryanodine receptors [27]. The size of Ca_v_1.2 clusters and their specific density in the plasma membrane depend on the type of *β* subunit expressed [31]. The distance between the termini of neighbor *α*_1C_ subunits varies from 67 Å with neuronal/cardiac *β*_1b_ to 79 Å with vascular *β*_3_. The highest density of Ca_v_1.2 clusters in the plasma membrane and the smallest cluster size were observed with *β*_1b_ present. Insight into molecular mechanisms defining the architecture and properties of Ca_v_1.2 clusters is important for better understanding of pathophysiology of the coupling between the Ca_v_1.2 activity and the induced responses in Ca^2+^ signal transduction.

## 3. Voltage- and Ca^2+^-Dependent Inactivation of the Ca_v_1.2 Calcium Channel

**Figure 3:**
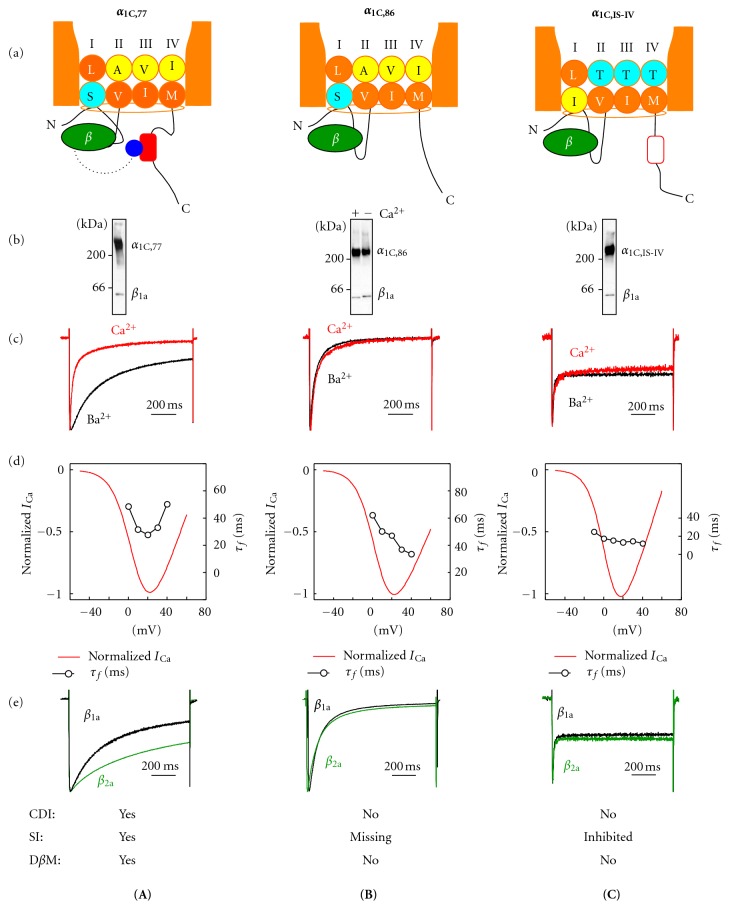
Molecular determinants of Ca_v_1.2 inactivation. Comparison of the wild-type Ca_v_1.2 (A) with the same channel deprived of CDI (B) and SI (C) determinants. The five horizontal panels show (a) arrangement of critical determinants of inactivation. ADSI is composed of conserved hydrophobic amino acids in a -2 position of S6 segments in repeats II, III, and IV (yellow circles: Ala, Val, and Ile, resp.) as well as Ser residue in -1 position of IS6 (cyan circle). The CaM-binding domain (CBD) of the *α*_1C_ C-tail is shown by a red rounded rectangle. A *β* subunit (green) binds to the *α*-interaction domain in the linker between repeats I and II, and, in a Ca^2+^-dependent manner, to the IQ-region of the *α*_1C_ subunit C-tail ([45], not shown). The distal structure of *β*_2_ (*β*_2_CED, blue ball) binds to the CBD [46]. (b) Evidence of coimmunoprecipitation of the indicated subunits. (c) Normalized traces of *I*_Ba_ (black) and *I*_Ca_ (red), and (d) voltage dependence of *I*_Ca_ (red) and time constant of FI (*τ*_*f*_, black) are presented to illustrate CDI in (A) and lack of CDI in (B) and (C). (e) Link between CDI and differential *β*-subunit modulation (D*β*M) of Ca_v_1.2. (A) Differential modulation of the *I*_Ba_ inactivation by *β*_1a_ (black trace) and *β*_2a_ (green trace) in the WT Ca_v_1.2. Disruption of CBD (*α*_1C,86_) eliminates CDI and SI targeted by CDI and D*β*M (B). Mutation of ADSI (*α*_1C,IS-IV_) removed CDI and fully inhibited SI so that the channel remains conducting for the duration of the depolarization stimulus (C).

 In the case of Ca_v_1.2 calcium channels, two different mechanisms are in control of Ca^2+^ current inactivation. One mechanism is driven by Ca^2+^ ions on the cytoplasmic side of the plasma membrane, whereas the other depends on transmembrane voltage. Experimentally, replacement of Ca^2+^ for Ba^2+^ as the charge carrier eliminates Ca^2+^-dependent inactivation (CDI) [32] so that the Ba^2+^-conducting calcium channels inactivate in a voltage-dependent manner by fast (FI) and slow (SI) mechanisms [33]. These three mechanisms of inactivation, FI, SI and CDI, and their major determinants are illustrated on Figure 3.

### 3.1. Ca^2+^-Dependent Inactivation and Calmodulin-Binding Domain of *α*_1C_

 There are several different determinants of CDI, but it was not until 1997 that the Ca^2+^-sensing site of CDI had been narrowed down to a stretch of the 80-amino-acid C-terminal sequence of *α*_1C_ encoded by exons 40–42 [34] (Figure 2) marked by red block in Figure 3(A) (panel a). A naturally occurring splice variation in this region in *α*_1C,86_ (Figure 3(B)) completely inhibited CDI as it is evident from the lack of deceleration of the current with Ba^2+^ as the charge carrier (panel c, black trace) as compared with *I*_Ca_ (red trace). Another characteristic feature of the inhibited CDI was lack of the current size dependence of *I*_Ca_ on voltage (Figure 3(B), panel d, open symbols) that stays in contrast to the U-shape dependence of the time constant of fast inactivation (*τ*_*f*_) on membrane potential in the wild-type Ca_v_1.2 (see Figure 3(A), panel d). Two distinct sequences, L and K, were identified within this 80-amino-acid stretch whose *α*_1C,86_-like mutations in the wild-type *α*_1C_ conform to the same characteristic features [35], suggesting the existence of two adjacent CDI sensors. One of them was outlined in the K region as the calmodulin- (CaM-) binding IQ motif [36] and, later on, the link of the IQ motif to CDI as the functional Ca^2+^-CaM binding site was confirmed in three independent studies [37–39] by the use of CaM mutants lacking affinity to Ca^2+^. Correspondingly, the LA motif was linked to CDI as apo-CaM binding site [40–42] endowed by the resting state of the channel. A single CaM molecule tethered to this Ca^2+^-dependent CaM-binding domain (CBD) of *α*_1C_ is the major Ca^2+^ sensor of the channel [43, 44].

 Splice variation of *α*_1C_ in CBD region of *α*_1C,86_ not only completely inhibits CDI, but also removes SI (Figure 3(B), panel c) and deprives the channel of differential sensitivity to *β*-subunit modulation (Figure 3(B), panel e) in spite of the fact that *β* remains associated with *α*_1C,86_ (Figure 3(B), panel b). This indicates that all three properties of the channel—CDI, SI, and *β*-subunit modulation—are linked together [48, 49].

### 3.2. Slow Inactivation

A number of evidences have been presented that amino acids confined to the distal part of S6 segments in *α*_1C_ play important role in SI [50–52]. Systematic study of this region [53] outlined the “*annual determinant of slow inactivation” *(ADSI) as a structure composed of four highly conserved amino acids of four transmembrane segments S6, constituting the cytoplasmic end of the pore (Figure 3(A), panel a). Their simultaneous mutation (S405I in IS6, A752T in IIS6, V1165T in IIIS6, and I1475T in IVS6) generates the *α*_1C,IS-IV_ channel. Analysis of the current kinetics of the *α*_1C,IS-IV_ channel showed tremendous acceleration of the rapidly inactivating component (*τ*_*f*_ ≤ 10 ms) that comprises about 50% of the total *I*_Ba_ (or *I*_Ca_) amplitude. Slow voltage-dependent inactivation of *α*_1C,IS-IV_ is fully inhibited, and the channel remains conducting for the duration of depolarization. Replacement of Ca^2+^ for Ba^2+^ as the charge carrier (panel c) did not change significantly this pattern of inactivation, while the analysis of voltage dependence of *τ*_*f*_ for the inactivating component of *I*_Ca_ through the *α*_1C,IS-IV_ channel (panel d) confirmed lack of CDI. The replacement of *β*_1a_ for *β*_2a_ (panel e) did not change inactivation of the *α*_1C,IS-IV_ channel current suggesting lack of differential *β*-subunit modulation, while the co-immunoprecipitation analysis (panel b) provided direct evidence of association between *α*_1C,IS-IV_ and *β*. 

 Taken together, results presented in Figure 3 suggest that there is a cross-talk between ADSI, CBD and *β*, supported by direct interactions between them and/or specific conformational folding of the constituents of the polypeptide bundle underlying the pore. Indeed, both the interaction of *β* with CBD and the importance of functional conformation were directly demonstrated in live cells expressing recombinant Ca_v_1.2.

### 3.3. Role of the *α*_1C_ C-tail Folding

**Figure 4:**
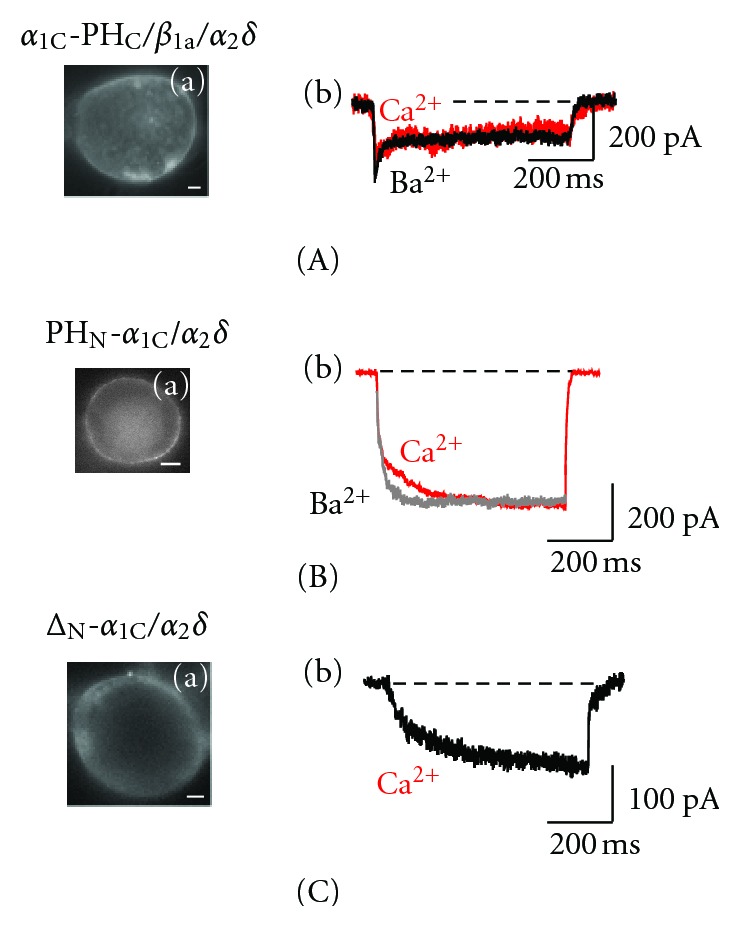
Differential role of the carboxyl- and amino-terminal tails of *α*_1C_ in Ca_v_1.2 inactivation. Shown are (a) epifluorescent images illustrating the plasma membrane targeting of the EYFP-labeled *α*_1C_ (scaling bars, 4 *μ*m) and (b) superimposed traces of the maximum *I*_Ba_ (black) and *I*_Ca_ (red) scaled to the same amplitude for *α*_1C_-PH_C_/*β*_1a_/*α*_2_ *δ* [21, 47], (A) PH_N_-*α*_1C_/*α*_2_ *δ* (B) [47], and Δ_N_-*α*_1C_/*α*_2_ *δ* (C) [47].

Quantitative voltage-dependent FRET microscopy combined with patch clamp in the live cell showed that the *α*_1C_ subunit C-terminal tail is subject to reversible voltage-gated conformational rearrangements [21, 47]. The anchoring of the *α*_1C_ C-tail to the inner leaflet of the plasma membrane via the pleckstrin homology (PH) domain fused to the C-terminus of *α*_1C_ (*α*_1C_-PH_C_) abolished this conformational rearrangement and inhibited both SI and CDI (Figure 4(A)) in a manner very similar to that observed with *α*_1C,IS-IV_ (Figure 3(C)). This modification limiting the mobility of the *α*_1C_ carboxyl terminus had major implication on Ca^2+^ signal transduction. CREB-dependent transcriptional activation associated with the activity of Ca_v_1.2 was completely suppressed in spite of robust *I*_Ca_ generated by the “C-anchored” channel in response to depolarization. Release of the *α*_1C_ C-tail by activation of PIP_2_ hydrolysis upon activation of phospholipase C fully restores all these deficient functions, including SI, CDI, and the effective coupling of *I*_Ca_ to the CREB-dependent transcription [21]. Thus, it is specific functional folding of the *α*_1C_ C-terminal tail that is crucial for inactivation. It is crucial for signal transduction because it is designed to cage the permeating Ca^2+^ in CaM attached to CBD and to effectively move this caged Ca^2+^ to downstream signaling targets associated with CREB-dependent transcription or cardiac muscle contraction [54]. Above all, this function occurs in tight coordination with extracellular stimuli activating the channel. In terms of signal transduction, SI is a lock on the inside of the channel that is released by the permeating Ca^2+^ to accelerate its closure and initiate the movement of the C-terminal tail [49].

### 3.4. Role of the *α*_1C_ N-Terminus

All the functions mentioned above depend also on the integrity of the *α*_1C_ N-terminus. Inactivation properties of the recombinant *α*_1C_/*β*/*α*_2_ *δ* channel are not greatly altered by structural changes of the proximal part of the *α*_1C_ N-tail, for example, by the fusion of a fluorescent protein [19, 21], by PH domain [47], or by alternative splicing of exons 1/1A generating the long isoform of *α*_1C_ [55]. The very first functional analysis of the effect of partial deletion of the *α*_1C_ N-terminus showed [56] that it is involved in inactivation while *β* prevents inhibition of the channel by the N-tail. Using FRET microscopy combined with patch clamp, we found that inactivation causes strong mutual reorientation of the *α*_1C_ and *β*_1a_ NH_2_-termini, but their distance vis-à-vis the plasma membrane is not appreciably changed [19]. This relative lack of mobility is conferred by *β* in a manner that facilitates the channel response to voltage gating. Experiments on uncoupling of the *α*_1C_ subunit N-terminal tail from the regulation of the channel were carried out in the absence of *β*. Anchoring of the *α*_1C_ N-tail in the inner leaflet of the plasma membrane via attached PH domain created conditions when PH_N_-*α*_1C_ and *α*_2_ *δ* were sufficient to generate a robust inward current (Figure 4(B)). This channel, however, is deprived of CDI and any voltage-dependent inactivation. Indeed, neither Ba^2+^ nor Ca^2+^ current has shown appreciable decay (see overlapped traces). Release of the *α*_1C_ N-tail upon PIP_2_ hydrolysis by activation of phospholipase C completely inhibited the *β*-deficient channel [47]. Similar properties, except a much slower activation of the current, were observed on deletion of the entire (but 4 amino acids) N-terminal tail of *α*_1C_ (Figure 4(C)). With either type of uncoupling of the *α*_1C_ N-terminal tail—whether through a deletion or by PM anchoring,—a delay in the activation of the whole-cell current appears to be associated with prolongation of the first latency. Single channel recordings revealed that deletion of the N-tail essentially stabilized the open state of the Δ_N_-*α*_1C_/*α*_2_ *δ* channel, which showed longer openings during long-lasting depolarization [47]. 

 Thus, CDI is mediated by CBD determinants of the *α*_1C_ C-tail, by the ADSI in the cytoplasmic pore region, and by the folding of the *α*_1C_ C- and N-termini. Calmodulin integrates these determinants, providing a Ca^2+^-dependent switch that terminates slow inactivation, releases the *α*_1C_ C-tail, and shuttles the associated Ca^2+^/calmodulin acting as an activating stimulus of the Ca^2+^ signal transduction [49].

### 3.5. Expression and Inactivation of Ca_v_1.2 in the Absence of the *β* and *α*_2_ *δ*

**Figure 5:**
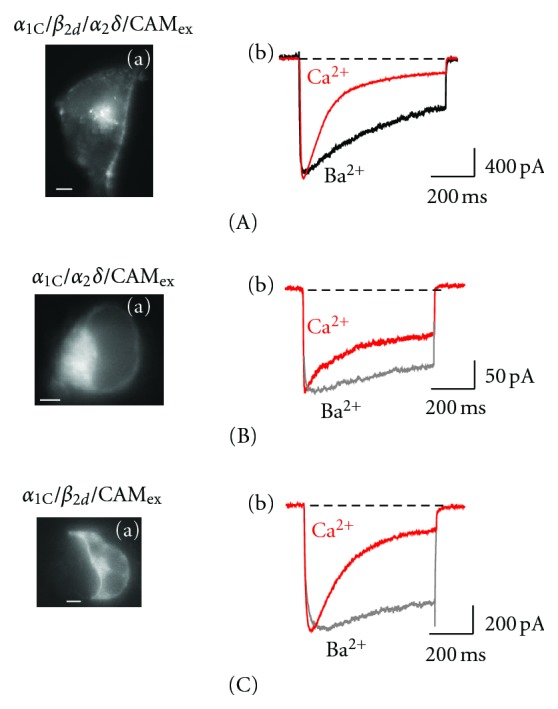
Activity of Ca_v_1.2 expressed in the absence of *β* or *α*_2_ *δ* subunits. Shown are (a) epifluorescent images illustrating the predominant PM localization of EYFP_N_-*α*_1C_ (scaling bars, 4 *μ*m) in COS1 cells and (b) superimposed traces of the maximum *I*_Ba_ (black) and *I*_Ca_ (red) scaled to the same amplitude for *α*_1C_/*β*_2d_/*α*_2_ *δ*/CaM_ex_ (A), *β*-free *α*_1C_/*α*_2_ *δ*/CaM_ex_ [18] (B) and *α*_2_ *δ*-free *α*_1C_/*β*_2d_/CaM_ex_ channel [57] (C).

Are the *β* and *α*_2_ *δ* subunits essential for the functional expression of the Ca_v_1.2 channel? The analysis of the effects of exogenous CaM (CaM_ex_) on the expression and properties of Ca_v_1.2 in the absence of either *β* [18] or *α*_2_ *δ* [57] clearly demonstrated that neither *β* nor *α*_2_ *δ* is essential. Overexpression of CaM_ex_ only slightly modifies the voltage gating of the *α*_1C_/*β*_2d_/*α*_2_ *δ* channel by shifting the voltage dependence of activation and inactivation towards more negative potentials, facilitating (but not accelerating) inactivation, and increasing the density of *I*_Ca_ approximately 2-fold [18]. CDI is retained, as it is evident from the effect of the replacement of Ca^2+^ for Ba^2+^ as the charge carrier that significantly increased the time course of inactivation of the current (Figure 5(A)). New understanding of the roles of *β* and *α*_2_ *δ* comes with the finding that CaM_ex_ renders expression and activity of the *α*_1C_ channel in the absence of *β* (Figure 5(B)) or *α*_2_ *δ* (Figure 5(C)), but not both of these auxiliary subunits. Although CaM_ex_ is structurally unrelated to *β* and *α*_2_ *δ*, it supports trafficking, CDI, and channel gating. Quantitative analysis showed that CaM_ex_ did not stimulate redistribution of *α*_1C_ in PM over the cytoplasm, but significantly enhanced plasma membrane targeting of *α*_1C_/*α*_2_ *δ* channels. On the other hand, CaM_ex_ did not enhance the relative distribution of the *α*_1C_/*β*_2d_ and *α*_1C_/*β*_2d_/*α*_2_ *δ* channels in the plasma membrane over the cytoplasm. Thus, depending on the auxiliary subunit present, CaM_ex_-supported channel activity of *α*_1C_/*β*_2d_ and *α*_1C_/*α*_2_ *δ* is under control of different mechanisms. In spite of that, the CaM_ex_-facilitated, single-auxiliary-subunit channels exhibit quite similar properties including significantly slower inactivation kinetics of the calcium current and a strong shift of the voltage dependence of activation and inactivation towards more negative potentials. Similar to the conventional *α*_1C_/*β*_2d_/*α*_2_ *δ* channels, these channels retain CDI and high sensitivity to dihydropyridine calcium channel blockers [18]. However, only the *α*_1C_/*β*/CaM_ex_ channel shows facilitation of the calcium current by strong depolarization prepulse [57] (data not shown).

 Because CaM associated with CBD is involved in CDI, it is clear that the effect of CaM_ex_ is mediated by different CaM-binding site(s). One of the potential candidates of such a site is present in the distal part of the *α*_1C_ N-terminal tail [58, 59]. It remains to be seen whether this site indeed plays an integrating role in the regulatory bundle of several molecular determinants supporting Ca_v_1.2 inactivation. Another possibility confines the role of CaM_ex_ to the activation of silent Ca_v_1.2 within the large clusters, where limited local availability of CaM may be the reason of the low fractional activity described in Section 2.5. Whatever the mechanisms associated with regulation of Ca_v_1.2 by CaM are, they seems to have little practical implication for use in medicine at this time exactly because CaM is a ubiquitous and multifunctional peptide that regulates many other cellular functions, while its presence in Ca_v_1.2 is vital for CDI. 

## 4. *β*-Subunit Modulation of Ca_v_1.2

Remarkable molecular variability of *β* subunits, reflected in altered inactivation properties of the differentially modulated Ca_v_1.2 [12, 60], exemplified in Figure 3(A) (panel e) presents a new opportunity for the development of innovative approaches to the treatment of the diseases associated with Ca^2+^ mishandling. Several recent observations provide a foundation for such an optimistic view. First, *β* subunits exhibit a tendency to form homo- and hetero-oligomers [11, 61] that was directly demonstrated by a variety of biochemical techniques in both native cells and in recombinant expression system. While an augmentation of *β* homooligomerization significantly increases the density of *I*_Ca_, heterooligomerization of *β*_2_ splice variants with other *β* subunits may also change the voltage-dependence and inactivation kinetics of Ca_v_1.2 [11]. The *β*-oligomerization is mediated by several molecular determinants and thus needs multiple interventions to be managed, for example, in case of pathogenic overexpression of *β*_2_. However, it seems to be more feasible to target *β*_2_ itself; molecular determinant of *β*_2_-specific slow and incomplete inactivation (see Figure 2(A), panel d) was identified [46] as the 40-amino-acid C-terminal determinant (*β*_2_CED) present in all 7 known naturally occurring *β*_2_ splice variants. Uncoupling of its Ca^2+^- and CaM-independent interaction with CBD (Figure 3(A), panel a) recovers the inactivation properties characteristic for *β*_1b_/*β*_3_-modulated Ca_v_1.2 exhibiting rapid and complete inactivation of *I*_Ca_, as it was shown in deletion experiments. In my view, such selective uncoupling of *β*_2_CED from binding to its receptor in CBD is a new attractive strategy to manage Ca^2+^ overload because other *β* subunits are not to be affected. Moreover, a cross-talk between Ca_v_1.2 and the nearest target Ca^2+^/CaM-dependent protein kinase II [62, 63] will be preserved.

## 5. Conclusions

This paper has demonstrated that we know how to accelerate inactivation of Ca_v_1.2 to *τ*_*f*_ less than 10 ms (Figure 3(C)), to deprive it from inactivation completely (Figures 4(B) and 4(C)), or to eliminate dependence of its expression from *β* or *α*_2_ *δ* without significant consequences for inactivation. We outlined the ultimate roles of the *α*_1C_ termini and CaM for inactivation, and yet none of these studies have brought us any closer to the ultimate goal of managing calcium mishandling associated with Ca_v_1.2 except of old and, unfortunately, not too selective calcium channel blockers. The only new feasible target is pathogenic *β*_2_ modulation of Ca_v_1.2, where effector-receptor interaction is established.

 In terms of molecular biology, Ca_v_1.2 is certainly among the most complicated regulatory systems known. Remarkable molecular diversity of each of the Ca_v_1.2 constituents gives rise to multiple genetic/splice variants of the channel that are subject to segregation into large and diverse clusters and to continuous functional change through homo- and hetero-oligomerization of *β* and other signaling components, not to speak about species, tissue, and developmental variability. We are surprised by the redundancy of the properties of multiple Ca_v_1.2 isoforms [64, 65] and are even more surprised when some of them, showing just “conventional” electrophysiological properties, turn out to be associated with a disease [13]. In looking for an explanation, our insight should not be intuitively focused just on the characteristics of the calcium current-voltage dependence, amplitude, and duration. The end response, such as spatial and temporal organization of CREB signaling events associated with specific Ca_v_1.2 isoform [66], and its competition with other (e.g., cAMP dependent) signaling mechanisms, or other Ca_v_1.2 isoforms present, may provide new ideas and open new frontiers for investigation of the roles of individual Ca_v_1.2 splice variants in normal and diseased cells and tissues.

## Abbreviations

ADSI: Annual determinant of slow inactivation
CaM: Calmodulin
CBD: Calmodulin-binding domain
CDI: Ca-dependent inactivation
ECFP: Enhanced cyan fluorescent protein
EYFP: Enhanced yellow fluorescent protein
FALI: Fluorophore-assisted light inactivation
FI: Fast inactivation
FRET: Fluorescent resonance energy transfer
GFP: Green fluorescent protein
SI: Slow voltage-dependent inactivation.
